# Highly Conductive, Anti-Freezing Hemicellulose-Based Hydrogels Prepared via Deep Eutectic Solvents and Their Applications

**DOI:** 10.3390/gels9090725

**Published:** 2023-09-06

**Authors:** Lisong Hu, Shishuai Gao, Lihui Zhao, Lili Dai, Daihui Zhang, Chunpeng Wang, Xuezhi Fang, Fuxiang Chu

**Affiliations:** 1 Research Institute of Subtropical Forestry, Chinese Academy of Forestry, Hangzhou 311400, China; hulisongyls@163.com; 2 National Engineering Laboratory for Biomass Chemical Utilization, Key Laboratory on Forest Chemical Engineering, SFA, Key Laboratory of Biomass Energy and Material, Institute of Chemical Industry of Forestry Products, Chinese Academy of Forestry, Nanjing 210042, China; gaoshishuai1006@163.com (S.G.); zlh17752199787@163.com (L.Z.); dai104735455@163.com (L.D.); wangcpg@163.com (C.W.); chufuxiang@caf.ac.cn (F.C.); 3 Co-Innovation Center of Efficient Processing and Utilization of Forest Resources, Nanjing Forestry University, Nanjing 210037, China

**Keywords:** hemicellulose, deep eutectic solvents (DESs), anti-freezing, conductivity, hydrogels

## Abstract

Hydrogels containing renewable resources, such as hemicellulose, have received a lot of attention owing to their softness and electrical conductivity which could be applied in soft devices and wearable equipment. However, traditional hemicellulose-based hydrogels generally exhibit poor electrical conductivity and suffer from freezing at lower temperatures owing to the presence of a lot of water. In this study, we dissolved hemicellulose by employing deep eutectic solvents (DESs), which were prepared by mixing choline chloride and imidazole. In addition, hemicellulose-based DES hydrogels were fabricated via photo-initiated reactions of acrylamide and hemicellulose with *N*, *N*′-Methylenebisacrylamide as a crosslinking agent. The produced hydrogels demonstrated high electrical conductivity and anti-freezing properties. The conductivity of the hydrogels was 2.13 S/m at room temperature and 1.97 S/m at −29 °C. The hydrogel’s freezing point was measured by differential scanning calorimetry (DSC) to be −47.78 °C. Furthermore, the hemicellulose-based DES hydrogels can function as a dependable and sensitive strain sensor for monitoring a variety of human activities.

## 1. Introduction

Hemicellulose is abundant in nature and is the second highest source of biomass resources after cellulose. It has been utilized in the manufacture of hydrogels, which are applied in heavy-metal adsorption, medication administration, wound dressings, and other fields due to its satisfactory biocompatibility and a huge number of active functional groups. For example, Hu and co-workers prepared xylan-based PAM hydrogels with high tensile and electrical conductivity from hemicellulose of the shell of *Camellia oleifera* Abel with the addition of MXene [1]. However, traditional hemicellulose-based hydrogels still suffer from several drawbacks, such as poor mechanical and electrical conductivity features. Therefore, it is crucial for building highly conductive hemicellulose-based hydrogels with adequate mechanical characteristics.

On the other hand, in addition to the explosive growth of portable electronic devices, the designing of environmentally friendly flexible electronic devices has recently garnered much research attention [2,3,4]. Hydrogels with 3D polymer networks are highly malleable, flexible, and biocompatible, which mean that they can be applied in touch screens, wearable devices, and flexible energy storage devices [5,6,7,8,9,10]. However, due to their poor electrical conductivity and lack of resistance at low temperatures, conventional hydrogels have been limited in their applicability. To overcome the drawbacks of conventional hydrogels, researchers have tried a variety of techniques, such as adding functional components to the hydrogel, modifying the ratio of reactants, and so on. To date, by adding conductive components to hydrogels [11,12,13,14,15], e.g., MXene, graphene, carbon nanotubes, polypyrrole, polyaniline, ions, etc., flexible conductive hybrid hydrogels with high electrical conductivity and excellent stability have successfully been synthesized. However, hybrid hydrogels may exhibit phase separation or inhomogeneous distribution as a result of incompatibility of the filler materials, which can somewhat reduce the mechanical and conductive capabilities of the hydrogels [16,17].

Some water-soluble conductive components, such as salts, ionic liquids, and organic acids, have been incorporated into hydrogel matrices to alleviate these problems [18,19,20]. When the temperature falls under the point of freezing, hydrophilic polymer hydrogels unavoidably freeze and become brittle, losing their original suppleness [21]. Therefore, the study of the hydrogels’ ability to withstand freezing is extremely significant to extend the applicability of hydrogels in extreme environmental tolerance. To add anti-freezing properties, hydrogels are currently manufactured by combining hydrophobic components with antifreeze chemicals. For example, hydrogels based on hydrophilic/oleophilic heterogeneous networks may withstand extremely low temperatures (−78 °C) [22]. Employing a binary H_2_O/glycol solvent, Rong and colleagues created a conductive self-healing hydrogel with consistent strain sensitivity in the −55.0 to 44.6 °C range in temperature [23].

One of the potential options for producing conductive hydrogels is the deep eutectic solvent (DES), a novel environmentally friendly solvent, because it exhibits low toxicity, it is easily available, and is of low cost [24]. DES is a two-part mixture comprising (1) hydrogen bond acceptors (HBA) like choline chloride (ChCl), thiocyanate, and others; and (2) hydrogen bond donors (HBD) involving urea, acrylamide, and others. Furthermore, the molar ratio and type of HBAs and HBDs are tunable, and therefore the physicochemical features of DES may be adjusted, making it widely applicable in polymer synthesis, compound extraction, organic solvents, harmful gas capture, and so on [25,26,27,28]. DESs are a new type of solvent that can not only dissolve lignocellulosic biomass (such as lignin, hemicellulose, and cellulose), but also provide electrical conductivity to the system. DESs and ionic liquids have comparable properties; they have the potential to create conductive hydrogels [29].

In this paper, in order to address the poor mechanical properties and limited functionalities of hemicellulose-based hydrogels, we developed a novel transparent hemicellulose-based hydrogel with high conductivity and anti-freezing properties using a simple two-step method. Specifically, this material was synthesized through a straightforward photo-initiated free-radical polymerization of acrylamide (AM) monomers in the presence of hemicellulose and deep eutectic solvents (DES). The role of each component in the hydrogel preparation was investigated by introducing DES to address the problems of difficult solubility of alkaline hemicellulose and poor mechanical and electrical conductivity of bio-based hydrogels. A comprehensive study of the physicochemical, mechanical, and electrical conductivity properties was carried out to reveal the function of the various parts. Hydrogen bonds between AM units within the hydrogel constitute the primary network. This PAM-DES-HC hydrogel possesses several attractive features, including stretchability, high toughness, transparency, and excellent electrical cycling stability. Finally, the potential of hemicellulose-based DES hydrogels for initial application as strain sensors to detect human activity was displayed as well. This study provides insight in designing hemicellulose-based functional hydrogels for various applications.

## 2. Results and Discussion

### 2.1. Preparation and Characterization of Hemicellulose-Based Hydrogels

**Figure 1:**
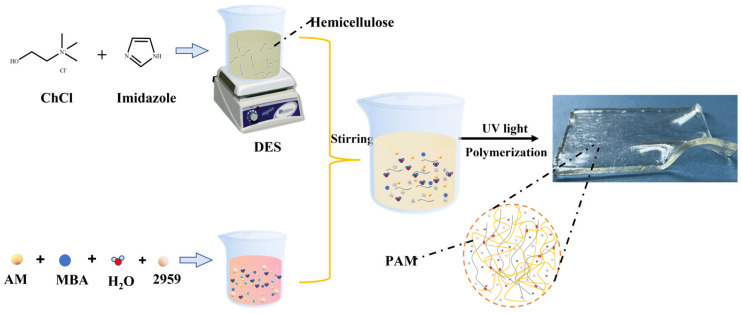
Schematic illustration of fabricating highly conductive and anti-freezing hemicellulosebased hydrogels via dissolving hemicellulose in ChCl-imidazole DES.

In this study, hemicellulose-based hydrogels were prepared via dissolving hemicellulose in ChCl-imidazole DES with high conductivity and anti-freezing properties. The hemicellulose was separated from *Camellia oleifera* Abel by adjusting the concentration of potassium hydroxide. The preparation process is shown in Figure 1. First, choline chloride and imidazole were combined and stirred at 80 °C till the mixture became clarified, homogenized, and translucent. The mixture was dried in a vacuum for 8 h to yield a solid deep eutectic solvent. Then, the hemicellulose was dissolved in ChCl-imidazole DES at 80 °C, and acrylamide, crosslinker agent MBA and photo-initiator 2959 were added. Finally, a photo-initiated polymerization process was applied. The prepared hydrogel was colorless and transparent.

**Figure 2:**
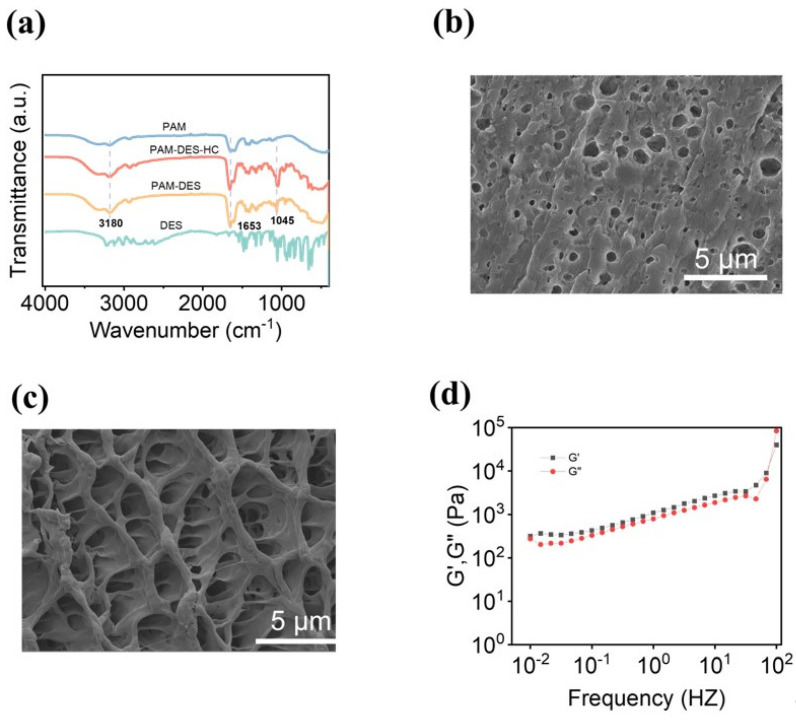
Characterization of the hydrogels. (**a**) FT-IR spectra of hemicellulose-based hydrogel and PAM hydrogel. (**b**) SEM image of the hemicellulose-based hydrogel. (**c**) SEM image of the PAM hydrogel. (**d**) Rheological properties of the hemicellulose-based hydrogels.

The structural features of the PAM, DES, PAM-DES, and PAM-DES-HC were studied with FT-IR spectroscopy to demonstrate the interactions that occur among the PAM, DES, and hemicellulose. The FT-IR spectra of the produced hydrogels are displayed in Figure 2a. The distinctive peaks of hydrogels (C=O) were at 1653 cm^−1^, which could be seen in PAM, PAM-DES, and PAM-DES-HC. The intensity of peaks at approximately 3180 cm^−1^ was ascribed to the stretching and bending vibration of O-H. The peak at 1045 cm^−1^ was ascribed to C-O-C stretching vibrations and -C-OH mixing vibrations in hemicellulose, respectively [30,31]. Vibrational bonds at 3200 cm^−1^ and 1800–880 cm^−1^ refer to a hydroxyl or amino group (N-H stretching the former and C-N^+^ symmetric stretching the latter); meanwhile, vibrational bands at 2990–2985 cm^−1^ referring to an alkyl group were also observed. The prepared hemicellulose-based DES hydrogels had a compact structure and a low number of pores compared to PAM hydrogels (Figure 2c). The G′ and G″ parameters were important for measuring dynamic rheological characteristics [32]. Figure 2d shows G′ > G″ values over the whole frequency from 0.01–100 Hz, which indicates that the hemicellulose-based DES hydrogels were elastic hydrogels. The viscoelastic modulus of the hemicellulose-based DES hydrogel increases with increasing scan frequency over the frequency scan range, indicating a frequency dependence of the viscoelastic modulus. The fundamental explanation for this might be the fact that the macromolecular chains did not instantly reorganize in the high-frequency range, causing them to stiffen, resulting in an increase in the moduli of G′ and G″, similar to the results of prior studies [33].

### 2.2. Mechanical Properties of the Hydrogels

**Figure 3:**
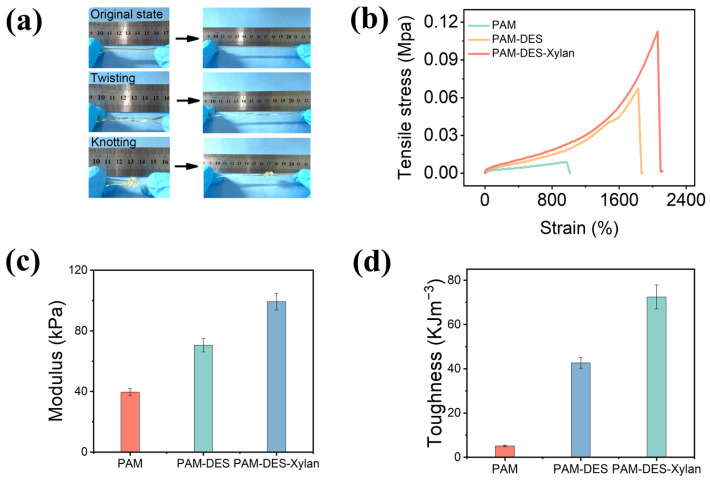
Tensile stress–strain curves, modulus, and toughness of the hemicellulose-based hydrogels. (**a**) Photographs of stretching, twisting, and knotting hemicellulose-based hydrogels. (**b**) Tensile stress–strain cures of the hemicellulose-based hydrogels. (**c**) Modulus of the hemicellulose-based hydrogels. (**d**) The toughness of the hydrogels.

The produced hydrogels can be stretched, twisted, and knotted as illustrated in Figure 3a, exhibiting their remarkable mechanical capabilities. Pure PAM hydrogels and PAM-DES hydrogels were examined under identical conditions to compare the mechanical characteristics of the hemicellulose-based hydrogels, and the results are depicted in Figure 3b−d. The tensile stress of the pure PAM hydrogels was 7.65 kPa, the strain was 977%, the modulus was 39.59 kPa, and the toughness was 5.09 kJ/m^3^. This study showed that DES may not only enhance hemicellulose dissolving and facilitate hydrogel production, but also offer hydrogel-enhanced mechanical characteristics. The explanation is that DES functions as a hydrogen bond acceptor and donor, and DES and PAM interact through hydrogen bonding to form a strong network system that improved the mechanical properties of the hydrogel, including its fracture stress of 112.73 kPa, strain of 2058.49%, modulus of 99.20 kPa, and toughness of 72.46 kJ/m^3^, which is consistent with the reported results [1]. When hemicellulose was added, the hemicellulose-based DES hydrogels had greater fracture stress and deformation, suggesting that the hydroxyl groups of hemicellulose interacted with PAM and DES through non-covalent bonds to improve mechanical properties.

**Figure 4:**
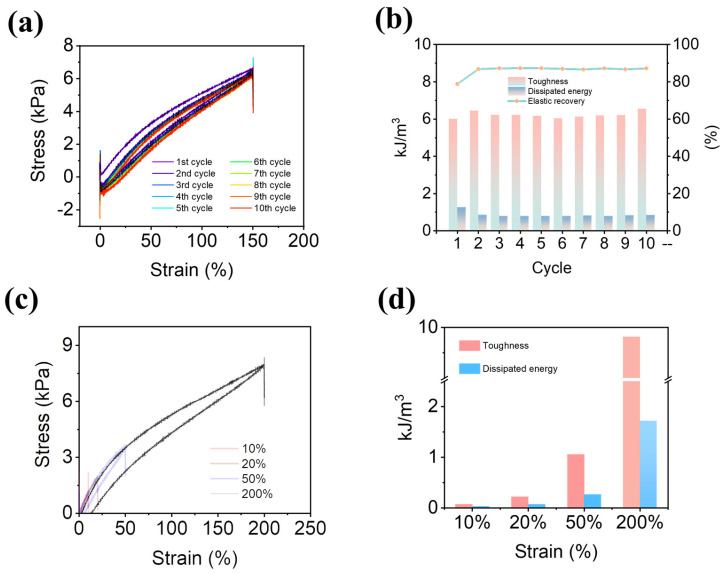
Strain cycling and energy dissipation in hemicellulose-based hydrogels. (**a**) Cyclic tests at the strain of 150%. (**b**) The toughness and dissipated energy at strain 10%, 20%, 50%, 200%. (**c**) The cyclic tests with increasing strain. (**d**) The calculated dissipated energy, dissipated ratio, toughness, and elastic recovery.

To investigate the elasticity and energy dissipation of hydrogels, loading–unloading experiments were carried out. The hydrogels were stretched 10 times at a set strain of 150%, as shown in Figure 4a, and the biggest amount of energy was dissipated for the first time with an energy dissipation of 1.31 kJ/m^3^. In addition, the hydrogels showed good elastic recovery, with an elastic recovery rate of 88%, as shown in Figure 4b. Loading–unloading experiments with various maximum stresses were performed to further study the energy dissipation of the hydrogels (Figure 4c). There was no break between the two loading sessions. When strain was increased, the energy wasted increased consistently from 6.2 kJ/m^3^ (100% of strain) to 34.2 kJ/m^3^ (200% of strain, Figure 4d).

### 2.3. Conductive Properties of the Hydrogel

**Figure 5:**
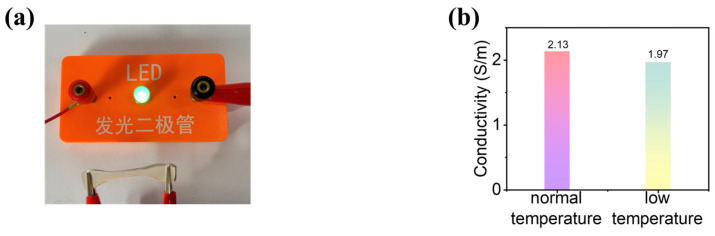
The electrical conductivity of hemicellulose-based hydrogels. (**a**) Photos of the luminance of LEDs (working voltage of 3.0 V) using hemicellulose-based hydrogels as the conductor. (**b**) The electrical conductivity of hemicellulose-based hydrogels at normal temperature (25 °C) and low temperature (−29 °C).

When the produced hydrogel cut in the shape of a dumbbell was connected to the circuit, as illustrated in Figure 5a, the PAM-DES-HC hydrogels could be used as part of an electric circuit to transmit the current that lights up an LED bulb, and the LED light showed significant brightness. This demonstrated that the prepared hydrogel had good electrical conductivity. Indeed, the electrical conductivity data from the prepared hydrogels showed that the hydrogel conductivity achieved 2.13 S/m at room temperature, with the conductivity decreasing marginally from room temperature to low temperature, with the electrical conductivity still reaching 1.97 S/m even at −29 °C, (Figure 5b). This was attributed to the fact that hydrogel networks consisting only of non-covalent interactions between each component, such as hydrogen bonding, and electrostatic interactions, may contribute to the formation of a DES system with unique properties and electrical conductivity. 

### 2.4. Anti-Freezing Property of the Hydrogels

**Figure 6:**
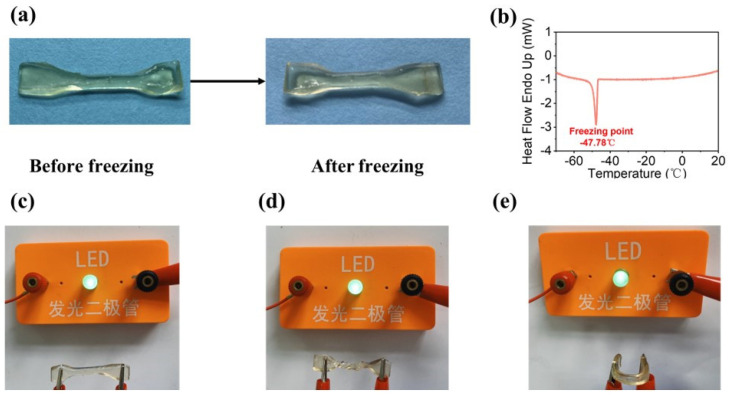
Anti-freezing properties of the hemicellulose-based hydrogels. (**a**) Photographs of the hydrogels before and after freezing in the −29 °C freezer. (**b**) DSC curve of the hydrogels. (**c**–**e**) Photographs of straightening, twisting, and bending the hydrogels after freezing at −29 °C, respectively.

The water content in hydrogels inevitably freezes in low-temperature environments, resulting in the loss of elasticity of hydrogels. Therefore, it is necessary to develop a hydrogel sensor with frost resistance to extend the operating temperature range of the sensor. After 24 h of freezing the hemicellulose-based hydrogel (Figure 6a) at −29 °C, revealed almost no difference, and the frozen hydrogel could be tested for mechanical behaviors such as tensile, twisting, and bending (Figure 6c–e). The hydrogels were analyzed for their anti-freezing properties by DSC (Figure 6b) and the hydrogel had an exothermic peak of water crystallization at −47.78 °C compared to −13 °C for the PAM hydrogel [34]. This was due to the fact that DES can lower the freezing point of water. These ions can interact with water molecules, limiting the formation of ice crystals, and enhancing the hydrogel’s antifreeze property. Furthermore, the prepared hydrogel had a stable and repeatable change in resistance, and the magnitude of the change in resistance at low temperatures was found to be higher than that at room temperature. The frozen hydrogel was tested for the change in hydrogel tensile-reciprocal resistance below 0 °C (−29 °C), and it was discovered that the prepared hydrogel had that change in resistance.

### 2.5. Application of PAM-DES-HC as Strain Sensors

**Figure 7:**
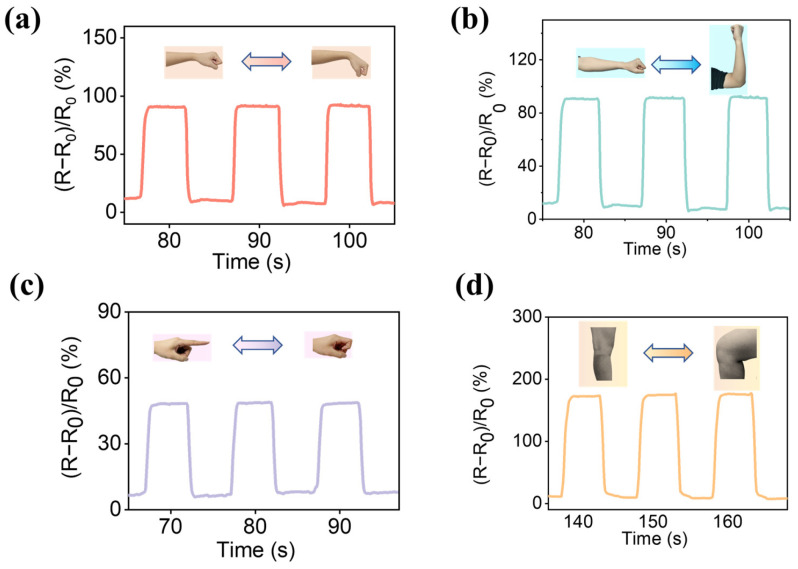
The strain sensor of the hydrogel’s application in human movements according to Δ*R* changed to monitor the experimental diagram of the preparation of hemicellulose-based hydrogels. (**a**) Wrist movements. (**b**) Elbow movements. (**c**) Finger movements. (**d**) Knee movements.

When DES-conducting hydrogels were stretched, the channel for DES ions to travel narrowed, increasing the resistance value and impeding the flow of HBA and HBD ions. It was, therefore, strain sensitive. Several applications for sensing human motion are displayed in Figure 7 to demonstrate the viability of this sensor in flexible electronic devices. To track human movement, the sensors were attached to various body areas of a 24-year-old female volunteer. DES hydrogel showed good cycle stability and durability. The resistive response signal in Figure 7a was reproducible and steady after repeated elbow flexion/extension movements. The relative resistance varied when the elbow was bent, but when the elbow was straightened again, the resistance reverted to its initial value. Notably, there was a positive correlation between angle and signal intensity when the change in the *R*/*R*_0_ signal at various angles was monitored. The relative resistance was constant while the finger was kept at a particular angle. At a fixed angle, the signal remained constant, and when the elbow was straightened, the signal was fully recovered. Numerous other body parts, such as the elbow and knee, were also subjected to similar motion detection. Figure 7b–d shows that various sensor extensions cause various changes in relative resistance. These results showed that hemicellulose-based DES hydrogels could be ideal sensors for applications in human motion detection.

## 3. Conclusions

In this work, we showed how to effectively produce multifunctional hemicellulose-based DES hydrogels using a straightforward technique. The prepared hydrogels have good mechanical properties, excellent electrical conductivity, and resistance to freezing with a conductivity of 2.13 S/m and still achieve a conductivity of 1.97 S/m at extreme temperatures (−29 °C). Hemicellulose and PAM interact via hydrogen bonding to increase the mechanical properties of the hemicellulose-based DES hydrogel, which had a fracture stress of 112.73 kPa, a strain of 2058.49% a modulus of 99.20 kPa, and a toughness of 72.46 kJ/m^3^. The hemicellulose-based DES hydrogel was the subject of an investigation into a flexible sensor that had high linearity, sensitivity, and stability across the strain range. The sensor had excellent repeatability and resistance-change stability. When the flexible sensor was fastened to the body, it was possible to accurately detect a variety of human motions. This work provides a new strategy to prepare hemicellulose-based hydrogels with enhanced mechanical properties and functionality compared to previous studies. It can serve to enlarge the potential application of hemicellulose-based hydrogels and inspire others to design multifunctional integrated wearable devices and smart bionic hydrogel soft robots.

## 4. Materials and Methods

### 4.1. Materials and Reagents

Acrylamide (AM), N, N′-methylenebisacrylamide (MBA), ammonium persulphate (APS), choline chloride, and imidazole were purchased from Aladdin Chemical Reagents Ltd. (Shanghai, China). Photo-initiator 2959 was obtained from Sigma-Aldrich Co., Ltd. St (Saint Louis, MO, USA). Hemicellulose was prepared from the shell of *Camellia oleifera* Abel (Jinhua, China) and the sugar analysis showed 95.82% xylose, 1.93%, 0.87%, and 0.69% glucose, mannose, and arabinose, respectively. All reagents were not further purified.

### 4.2. Preparation of Hemicellulose-Based Hydrogels

#### 4.2.1. Preparation of ChCl-Imidazole DES (Solution A)

Imidazole and choline chloride were dried and stored in a desiccator for future use. The heating process was used to prepare DES. In a round bottom flask, choline chloride 52.36 g and imidazole 59.57 g (choline chloride: imidazole 7:3 (mol/mol)) were weighed and heated at 80 °C with an electromagnetic stirrer (RT10, IKA, Staufen, Germany) at a 50 rpm rate until the mixture became a clear, homogeneous, and transparent liquid. The mixture was then heated for another 30 min, dried under vacuum for 8 h, and a solid product of ChCl-imidazole DES was obtained according to the previous report with modification [35]. The solid product was melted into liquid at 80 °C and named solution A for subsequent uses.

#### 4.2.2. Configuration of PAM Prepolymers (Solution B)

A total of 12 g of AM was dissolved in 30 g of water and stirred at 25 °C with an electromagnetic stirrer (RT10, IKA, Staufen, Germany) at a 100 rpm rate for 2 h. Then, 2.4 g of hemicellulose (prepared from the shell of *Camellia oleifera* Abel) was added to the AM solution and the mixture was stirred with an electromagnetic stirrer (RT10, IKA, Staufen, Germany) at room temperature for 30 min. Finally, MBA was added to the above solution and stirred with an electromagnetic stirrer (RT10, IKA, Staufen, Germany) at 25 °C for 30 min till a translucent and clear solution was obtained and stored in a freezer cabinet at 4 °C. This solution, named solution B, was set aside for polymerization.

#### 4.2.3. Fabrication of Hemicellulose-Based Hydrogels 

To synthesize a hemicellulose-based DES hydrogel, the following procedure was conducted: solution A, 3 mL, and solution B, 9 mL were mixed together and stirred at ambient temperature for 30 min. Then, photo-initiator 2959, 0.27 g, was added into the mixture, and stirred with a magnetic stirrer (RT10, IKA, Staufen, Germany) at ambient temperature for 30 min, poured into a PTFE mold, and placed in the reactor. Afterwards, the mixture was put in a light curing oven (36 W, 365 nm wavelength) and kept for 120 s.

### 4.3. Characterizations

FT-IR analysis was performed on samples ranging from 4000 to 400 cm^−1^ with a spectral resolution of 4 cm^−1^. The spectra were recorded on a single reflection attenuated total reflection (Nicolet iS50, Thermo Fisher Scientific, Waltham, MA, USA). A scanning electron microscope (SEM) (Gemini 450, Zeiss, Oberkochen, Germany) was used to characterize the hydrogels. Cured hydrogel samples placed in the circuit were frozen at −40 °C and then freeze-dried for 24 h. Next, the samples coated with 5 nm of gold were sliced and manually fractured in cross-section.

### 4.4. Mechanical Properties Testing of the Hydrogels

Tensile tests were conducted using universal testing equipment (UTM2503, SANS, Shenzhen, China) and a 20 N load cell on dumbbell-shaped hydrogels (4 mm × 25 mm). By using linear fitting (stress–strain), the modulus of a hydrogel was calculated, with the slope indicating the modulus. The total area curve was used to calculate toughness. Calculations for elastic recovery, dissipated energy, and dissipated ratio were performed in accordance with earlier research by Jian et al. [36]. Elastic recovery (*E*) was determined by employing the following equation to assess the mechanical properties of cyclic loading–unloading:

(1) $$E=\frac{\varepsilon_{max}-\varepsilon_{min}}{\varepsilon_{max}},$$

where *ε_max_* represents the maximum strain in the loading circle and *ε_min_* represents the strain at the point in the loading circle when the tensile stress is zero.

The dissipated energy (Δ*U_i_*) was calculated as follows:

(2) $$\Delta U_{i}=\oint \sigma d\varepsilon ,$$

where Δ*U_i_* is dissipated energy, *σ* and *ε* are tensile stress and strain, respectively.

The dissipated ratio (*η*) was used to measure the rates of dissipating energy of the materials.

The dissipated ratio was measured via the following equation:

(3) $$U_{i}=\int_{0}^{\sigma_{max}}\sigma d\varepsilon ,$$

where *U_i_* is the elastic energy of the materials during the tensile stress from *σ* = 0 to *σ_max_* in the loading circle. The dissipated ratio (*η*) was calculated as follows:

(4) $$\eta =\frac{\Delta U_{i}}{U_{i}}.$$

Water content was calculated according to the following equation:

(5) $$\omega \left(H_{2}O\right)=\frac{m_{s}-m_{d}}{m_{s}}\times 100\%,$$

where *m_d_* is the sample’s weight after drying and *m_s_* is the sample’s weight at equilibrium.

### 4.5. Testing of the Electrical Conductivity of Hydrogels

#### 4.5.1. Electrical Conductivity of Hydrogels (*σ*)

The evaluation of hemicellulose-based hydrogels was performed using an electrochemical workstation (CHI-660E, Chen Hua, Shanghai, China). According to the equation, the conductivity was determined as follows:

(6) $$\sigma =\frac{L}{R\times S},$$

where *σ* (S/m), *L* (m), *R* (Ω), *S* (m^2^) are the conductivity, length, resistance, and contact area of the samples, respectively.

#### 4.5.2. Hydrogel Resistance Variation

An LCR meter (TH2830, Tong Hui, Shanghai, China) was used to capture the hydrogel resistance signals using the following formula:

(7) $$\frac{R-R_{0}}{R_{0}}\times 100\%,$$

where *R* (Ω) are the resistance of the sample at different strains; *R*_0_ is the sample resistance at the 0% strain.

### 4.6. Freezing Resistance of Hydrogels

A differential test scanning calorimeter (DSC 214 Polyma, NETZSCH, Selb, Germany) was used to measure the hydrogels’ ice crystallization temperature in the range of −80 °C to 25 °C at a temperature-decreasing rate of 5 °C/min. To measure the freezing resistance of the hydrogels, the samples were sealed and placed in a refrigerator at −29 °C for 12 h and then removed and immediately observed to determine whether they remained elastic and to demonstrate the performance of hydrogels such as conductivity, mechanical properties of stretching, twisting and knotting.

### 4.7. Rheological Testing

On a parallel plate rheometer (AR 2000, TA Instruments, New Castle, DE, USA), the hydrogels’ dynamic rheological characteristics were assessed. Before being deposited onto a Brookfield DVIII instrument plate, the samples were first dissolved in a 1wt% NaOH solution at 25 °C and agitated until a homogeneous and stable clear solution was created. To prevent the water from evaporating from the solution, the borders of both parallel plates were sealed with silicone oil. With shear rates ranging from 10^−2^ to 10^3^/s and storage (G′) and loss (G″) moduli measured at oscillatory strain rates of 10^−2^ to 10^2^/s, specific strain values were set to ensure that the shear tests were carried out within a linear viscoelastic range of dynamic storage and loss moduli.

## Data Availability

The authors declare that they have no known competing financial interests or personal relationships that could have appeared to influence the work reported in this paper.
